# Oral colon-targeting core–shell microparticles loading curcumin for enhanced ulcerative colitis alleviating efficacy

**DOI:** 10.1186/s13020-021-00449-8

**Published:** 2021-09-22

**Authors:** Chen Zhang, Zhejie Chen, Yanan He, Jing Xian, Ruifeng Luo, Chuan Zheng, Jinming Zhang

**Affiliations:** 1 State Key Laboratory of Southwestern Chinese Medicine Resources, Chengdu University of Traditional Chinese Medicine, Chengdu, 611137 PR China; 2 State Key Laboratory of Quality Research in Chinese Medicine and, Institute of Chinese Medical Sciences, University of Macau, Macau, 999078 PR China; 3 Oncology Teaching and Research Department, Hospital of Chengdu University of Traditional Chinese Medicine, Chengdu, 610072 PR China; 4 Present Address: College of Pharmacy, Chengdu University of Traditional Chinese Medicine, Chengdu, 611137 PR China

**Keywords:** Coaxial electrospray, Curcumin, Zein, Microparticles, Ulcerative colitis

## Abstract

**Background:**

The oral colon-targeting drug delivery vehicle is vital for the efficient application of curcumin (Cur) in ulcerative colitis (UC) treatment because of its lipophilicity and instability in the gastrointestinal tract.

**Methods:**

The core–shell microparticle (MP) system composed of eco-friendly materials, zein and shellac, was fabricated using a coaxial electrospray technique. In this manner, Cur was loaded in the zein core, with shellac shell coating on it. The colon-targeting efficiency and accumulation capacity of shellac@Cur/zein MPs were evaluated using a fluorescence imaging test. The treatment effects of free Cur, Cur/zein MPs, and shellac@Cur/zein MPs in acute experimental colitis were compared.

**Results:**

With the process parameters optimized, shellac@Cur/zein MPs were facilely fabricated with a stable cone-jet mode, exhibiting standard spherical shape, uniform size distribution (2.84 ± 0.15 µm), and high encapsulation efficiency (95.97% ± 3.51%). Particularly, with the protection of shellac@zein MPs, Cur exhibited sustained drug release in the simulated gastrointestinal tract. Additionally, the in vivo fluorescence imaging test indicated that the cargo loaded in shellac@zein MPs improves the colon-targeting efficiency and accumulation capacity at the colonitis site. More importantly, compared with either free Cur or Cur/zein MPs, the continuous oral administration of shellac@Cur/zein MPs for a week could efficiently inhibit inflammation in acute experimental colitis.

**Conclusion:**

The shellac@Cur/zein MPs would act as an effective oral drug delivery system for UC management.

## Introduction

Ulcerative colitis (UC) is a chronic, recurrent, and debilitating inflammatory disease which causes marked disturbance in colon inflammatory homeostasis and severe disruption in intestinal barrier function, affecting millions of people worldwide. it cannot be completely cured and must be managed throughout life [1]. Presently, the drug treatment of UC depends on amino salicylate, corticosteroids, immune suppressants, or antibiotics to control tissue inflammation, resulting in mucosal healing. However, the long-term intake of these drugs can cause serious adverse effects for patients [2, 3], such as acute pancreatitis, imbalance of the intestinal flora, and osteoporosis. Thus, it is very important to develop therapeutic agents with high efficacy and fewer adverse effects [4, 5].

Curcumin (Cur) with a high oral administration safety is a natural bioactive polyphenol derived from the turmeric rhizome (*Curcuma longa* L.) [6], generally recognized as safe by the FDA. Moreover, same pre-clinical studies have provided compelling evidence for the use of Cur to ameliorate UC because of its excellent antioxidant and anti-inflammatory properties [7, 8]. Hence, Cur as a multi-targeted and cost-effective agent has attracted much attention in UC prevention and treatment. Despite these advantages, its further application for UC treatment has been severely restricted because of its strong hydrophobicity, high intestinal metabolic rate, instability in the gastrointestinal tract, and rapid excretion from the body [9]. To circumvent these issues, multifarious oral drug delivery systems, including pellets, microparticles and nanoparticles, have been exploited to deliver Cur directly to the colitis tissues [10–15].

Efficient local tissue targeting and rapid drug release at the colitis tissue are the main challenges in UC treatment. Although some large-size drug delivery systems (e.g., pellets, tablets or capsules) facilitate efficient drug encapsulation, the colon delivery efficiency was commonly limited, since the particles larger than 200 µm are strongly subjected to diarrhea [16]. Additionally, free drug released from large conventional formulations cannot be efficiently adsorbed because of the overexpressed glycoprotein and cytochrome P450 on the surfaces of inflamed intestinal cells. Thus, developments of micro-/nano-sized carriers may not only induce the drugs accumulate in the site of inflamed colon by EPR effect, but also improve drug efficacy and reduce the side effects for therapeutic drugs [17]. 

In recent years, the oral administered microparticle (MP) system has emerged as one of the best approaches for controlled drug delivery at the specific site of inflammation [18]. Previous studies found that because of the pathological changes in UC tissues, including the damage of colonic barriers and the accumulation of immune cells, MPs with diameters < 10 μm can preferentially penetrate and accumulate into the colitis tissue [19]. Additionally, Coppi et al [20] reported that the optimum particle size should be 4–15 μm to improve localization and increase the residence time of drug at the inflammation site. Compared to nanoparticles, MPs exhibited the higher capacity to resist protein/enzyme adsorption, thereby avoiding the harsh environment of gastrointestinal tract [21]. Consequently, various orally administered MPs have been developed and have achieved increased drug accumulation in the colitis tissue, improved therapeutic outcomes, and reduced the occurrence of adverse effects.

Zein, belonging to a family of naturally alcohol-soluble prolamins from corn, has received increasing attention from researchers for drug delivery and tissue engineering because of its inherent biocompatibility and degradability [22]. During the previous few decades, various carriers [23, 24], including nano/microparticles, nano/microcapsules, films, nanofibers, and hydrogel, have been investigated using zein for drug delivery. More importantly, the insoluble zein in an aqueous solution, resistance to stomach acidic pH and the enzyme makes it an ideal carrier for oral administration. For instance, aceclofenac, a water-insoluble nonsteroidal anti-inflammatory drug that generally induces gastric mucosal injury, could be significantly delayed its release in pH 1.2 simulated gastric fluid, after loading in zein MPs cross-linked with glutaraldehyde [25]. However, zein still can be rapidly degraded in the presence of intestinal enzymes. Thus, the efficiently localized drug delivery by zein highlights the need for a secondary material to prevent drug release in advance in the intestinal tract. Shellac, a resin secreted by the female lac beetle, is a potentially important material in colon-targeting carrier development because it is insoluble in the stomach and soluble in a neutral environment [26, 27]. Shellac-coated tablets are ubiquitous in the pharmacy; meanwhile, research on shellac-related colon-targeted drug delivery carriers, such as shellac-coated pectin beads [28], shellac nanofiber [29], and shellac coating nanocomposite [26], is ongoing.

**Fig. 1:**
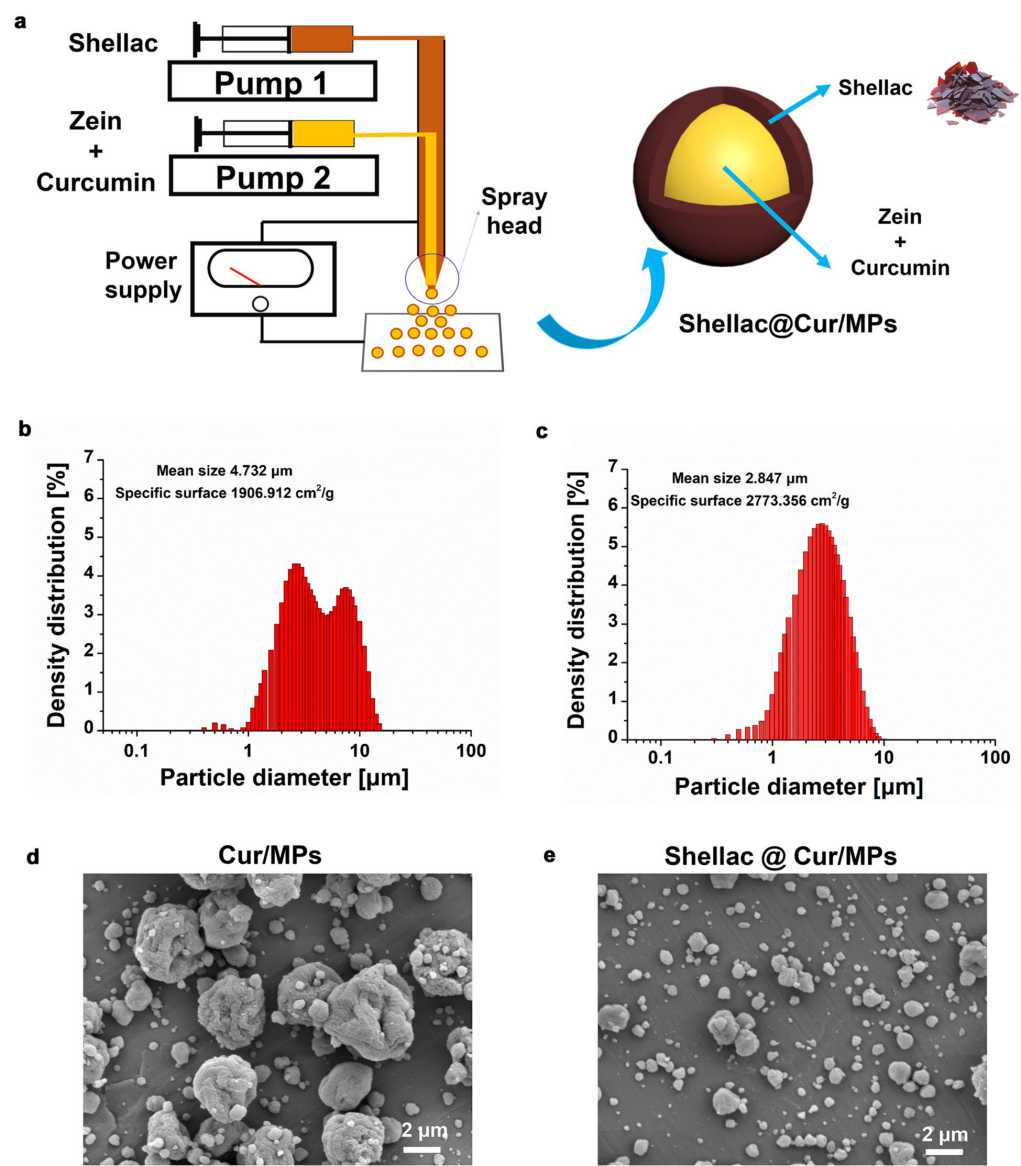
**a** A schematic illustration for the fabrication of Shellac@Cur/MPs; Size distribution of Cur/MPs (**b**) and shellac@Cur/MPs (**c**) by DLS determination. SEM images of Cur/MPs (**d**) and shellac@Cur/MPs (**e**), respectively

Here, in comparison with emulsification as the common MPs fabrication method, we applied electrospray, an alternative, simple, one-step method with high encapsulation efficiency and controllable drug release profiles [30], to encapsulate Cur into zein. The process of MP preparation is based on a conical jet formed by electrostatic force. That is, a liquid meniscus flows out from the tip of a capillary in a high-voltage electric field and finally breaks into preliminary droplets. Meanwhile, the coaxial electrospray (CES) technology was used to form MPs with a core–shell structure using the coaxial jet of two immiscible liquids [31]. Recently, Xu et al loaded artemether [32] and Cur [33] in PLGA MPs mediated by CES for high encapsulation efficiency and sustained drug release. Therefore, we produced core–shell structured zein MPs loading Cur with shellac-coated (shellac@Cur/MPs) by CES. During this approach, the mixture of zein and Cur solution and shellac solution acted as the inner and outer liquid flow in the coaxial needle (Fig. 1a). After optimizing the operating parameters, such as the applied electric voltage, the inner and outer liquid flow rates, and the coaxial needle configurations, shellac@Cur/MPs could be formed with uniform size and high drug loading efficiency (DLE). Then, compared with the unloaded Cur and zein MPs loading Cur without shellac protection, both *in vitro* physicochemical characterization and *in vivo* anti-UC experiments were performed to evaluate the advantages of shellac@Cur/MPs for UC treatment.

## Materials and methods

### Chemicals and reagents

Dextran sulfate sodium (DSS, 36–50 kDa) was purchased from Seebio^®^ Biomedicals (Xian, China). Shellac (purity ≥ 95%, batch no. BCI-16072505) was procured from Shanghai Yuan Ye Biotechnology Co., Ltd. (Shanghai, China). The zein protein (purity ≥ 98%) was purchased from Tokyo Chemical Industry (Tokyo, Japan). Curcumin (purity ≥ 98%) and IR780 were purchased from Sigma-Aldrich (St. Louis, MO, USA). Ethanol was procured from Aladdin (Shanghai, China). The myeloperoxidase (MPO) kit, interleukin-6 (IL-6) kit, interleukin-1β (IL-1β) kit, and tumor necrosis factor-alpha (TNF-α) kit, were supplied by MultiScience (Lianke) Biotech Co., Ltd. (Zhejiang, China).

Male BALB/c mice (22–25 g) were obtained from SPF Biotechnology Co., Ltd. (Beijing, China). The mice were kept under standard conditions and freely supplied with food and distilled water. All animal experiments were performed in accordance with the protocol approved by the Animal Ethics Committee of Chengdu University of Traditional Chinese Medicine.

### Preparation of Cur/MPs and shellac@ Cur/MPs

The Cur/MPs and shellac@ Cur/MPs were prepared with CES technology using an electrospray–electrospinning system (TL-OMNI, TONGLI, Shenzhen, China), equipped with two pumps, on power supply (high-voltage 0–45 kV), a concentric spray head, and a product collector. For CES process, the inner blending solutions comprised 9% zein (w/v) and 1% Cur (w/v) in 80% ethanol solution and the outer solution comprised 10% (w/v) shellac in ethanol. By contrast, Cur/MPs without shellac coating were prepared using a single axial electrospray process, using the 9% zein (w/v) and 1% Cur (w/v) blending solution.

For CES process, the work solutions were processed by either a single stainless-steel needle (0.9 mm inner diameter) or two concentric stainless-steel needles (outer diameter: 1.4 mm, inner diameter: 0.6). The solution was placed in the plastic syringe placed on the digital pump and pumped in at a steady flow via the PTEE line connected to the needle. The processed microparticles was collected on a stainless-steel plate connected to the cathode of the power supply. The collected fine particles were jetted down to the negative voltage on the stainless-steel iron. After several pre-experiments, the solutions were maintained at a shell and the core fluid flow rate of 0.2 and 0.8 mL/h by the twin syringe pump, respectively. The applied positive voltage and the applied negative voltage were 15 and 25 kV, respectively. The distance between the needle and the collector was set at 15 cm. The shellac@Cur/MPs were collected on an aluminum plate.

In the same method, we prepared the Cur/MPs using a single-fluid electrospraying approach for the comparative study. In this process, the Cur and zein blending solution at the same concentration except that the coaxial needle was replaced by a single axial needle, without shellac solution. The flow rate of the core fluid was set at 1.0 mL/h.

### Physicochemical characterization of MPs

The size distribution of Cur/MPs and shellac@Cur/MPs were measured via dynamic light scattering (DLS) with a Laser Particle Analyzer PSA 1190 (Anton Paar, AT). The morphology of the MPs was observed by a JEOL JEM-2100 scanning electron microscope (SEM) (Tokyo, Japan).

Fourier transform infrared (FTIR) analysis was performed using a Spectrum 100 FTIR spectrometer (PerkinElmer, Billerica, USA) over the range of 500–4000 cm^−1^ and at a resolution of 2 cm^−1^. X-ray diffraction (XRD) analysis was performed to assess the physical form of the components in the MPs. XRD spectra were recorded using a Cu Ka-ray at 45 kV and 40 mA ranging from 5° to 70° in an XRD instrument (X’Pert PRO, PANalytical, Netherlands). The samples for both FTIR and XRD analyses were shellac flakes, zein, Cur, and the physical mixture of these three components (shellac + zein + Cur), Cur/MPs and Shellac@Cur/MPs, respectively.

### Drug loading efficiency and drug encapsulation efficiency

To determine drug encapsulation efficiency (DEE) and the drug loading efficiency (DLE) of Cur in MPs, the amount of Cur was estimated with SHIMADZU DGU-20A 3R/5R HPLC (SHIMADZU, Japan) equipped with a reverse phase C_18_ column (250 × 4.6 mm, 5 μm) at a maximum absorbance of 425 nm. The mobile phases for Cur determination were composed as acetonitrile: 0.2% acetic acid (65:35, v/v). The flow rate was 1 mL/min. DLE and DEE of Cur in the MPs were calculated using the following equations (1) and (2):

1 $${\text{DLE\% }}\,{ = }\,\left( {\text{Weight of Cur in MPs}} \right){/}\left( {\text{Weight of Cur loaded MPs}} \right) \times {\text{100\% }}$$

2 $${\text{DEE\% }}\,{ = }\,\left( {\text{Weight of Cur in MPs}} \right){/}\left( {\text{Weight of initially feed Cur}} \right) \times {\text{100\% }}$$

### In vitro* release profiles of Cur from MPs*

*In vitro* Cur release profiles from Cur/MPs or shellac@Cur/MPs were carried out via a dialysis method. Three types of drug release media with different pH values, i.e., artificial colonic fluid (pH 7.8), artificial intestinal fluid (pH 6.8) and the simulated gastric fluid (pH 1.2), were applied to simulate the digestive tract environment. Both Cur/MPs and Shellac@Cur/MPs suspended in PBS (equal to 400 µg of Cur) were introduced into a regenerated cellulose dialysis bag (molecular weight cut-off 10000 Da), and the closed bag was put into a centrifuge tube with 40 mL of the releasing medium at 150 rpm and 37 °C. At specific time intervals, 1 mL outer solution was withdrawn for measurement and replaced by the same volume of fresh releasing medium; 1 mL of acetonitrile was added in 1 mL of the collected supernatant, and the drug was extracted with ultrasonography treatment for 30 s. The solution was centrifuged at 12,000 rpm for 5 min to remove the impurities. Finally, the Cur amount in the outer solution was determined using the HPLC method as mentioned above. All the procedures were performed in triplicate.

### In vivo* distribution and retention of MPs*

The colorectal distribution and retention of zein MPs and shellac-coated zein MPs after intragastric administration were studied using *in vivo* fluorescence imaging in a colitis mouse model. The near-infrared fluorescent probe IR780 was loaded into MPs as the fluorescence label, using a similar preparation approach as that of Cur/MPs. Briefly, male BALB/c mice were fed water containing DSS (2.5 %, w/v) for three consecutive days to produce colitis. Thereafter, the same volume of IR780, IR780/MPs, and shellac@IR780/MPs suspension in PBS containing 0.15 mg/mL of IR780 was intragastrically administrated into colitis mice. The abdominal fur was trimmed using a hair clipper to reduce interference during *in vivo* imaging. The animals were euthanized at 3, 6, 12, or 24 h post-administration and evaluated with NIR imaging using AniView 100 live imaging system (BLT, Guangzhou, China) at wavelength values of excitation and emission of 770 and 830 nm, respectively. After *in vivo* images were taken at 24 h, the mice were sacrificed, and the distal colons were collected and analyzed again with NIR imaging.

### In vivo* anti-UC efficacy*

The UC mice model was established by giving DSS (3 %, w/v) in drinking water ad libitum. After 3 days of DSS treatment, the mice were randomly divided into four groups (six mice per group): UC model group, free Cur group, Cur/MPs group, and Shellac@Cur/MPs group, in which 5 mg/kg of Cur was administered every day for 7 days. The UC model group mice were only orally administered fresh water. Moreover, mice without DSS treatment were applied as the controls.

During the experimental period, the body weights of mice were recorded every day. The disease activity index (DAI) was recorded as the mean value of the following parameters: normal stool (0), soft stools (1), soft stools and slight bleeding (2), loose stools and slight bleeding (3), and gross bleeding (4). On day 10, the mice were sacrificed with CO_2_ euthanasia, and the distal colons were collected. The MPO levels in the colon were examined using a commercially available MPO kit as per the manufacturer’s instructions. Colon segments in each group were fixed in paraformaldehyde, embedded in paraffin, sectioned into slices (5 μm), and subjected to H&E staining and PAS staining. The amount of the main pro-inflammatory cytokines (IL-1β, IL-6, and TNF-α) in the colon tissue was detected using the corresponding commercially available ELISA kit as per the manufacturer's instructions.

### Statistical analyses

All the data are expressed as mean ± standard deviation (SD) values and were analyzed with GraphPad Prism5.0 (GraphPad Software, San Diego, USA). One-way ANOVA with Bonferroni test was used to examine differences among the groups. P-value of < 0.05 was considered to indicate a significant difference.

## Results

### Fabrication of MPs

During the CES process, some key parameters, such as the flow rate, applied voltage, working fluid solvent, and concentrations of Cur and zein materials, have been critically optimized for forming a stable coaxial cone-jet model. Commonly, two most studied process parameters, including the applied voltage and the flow rates, contribute to droplet size, cone-jet stability, shell thickness, and other performance characteristics of the process. Briefly, we previously observed the mode transition in CES, from the dripping mode, coning mode in the spindle, stable cone-jet mode, to multi-jet mode, with an increase in the applied voltage. After optimization, the electrical voltage was fixed at 21 kV, generating the round microspheres. Additionally, we found that the droplet size depends on the flow rate of the working liquid. The decreased flow rate was of benefit to diminish the size of MPs. Under the selected conditions, the shellac@Cur/MPs were produced from a shell and core fluid flow rate of 0.2 and 0.8 mL/h. When the inner and outer solutions flow through the coaxial needle, they eventually form small droplets under the influence of the applied voltage. These tiny droplets dry in flight before reaching the collector, and the dried MPs can be collected.

### Physicochemical characterizations of MPs

A previous study has revealed that either small or oversize particles will be easily excreted from the body due to diarrhea [34], one of the most usual symptoms in UC patients. Therefore, particle size distribution is critical for MPs that directly impact the stability, biodistribution, and mucoadhesive properties of MPs. As Table 1 summarizes, the DLS measurements indicated that the average hydrodynamic diameter of Cur/MPs and Shellac@Cur/MPs was 4.73 and 2.84 µm, respectively. The surface morphology were confirmed as smooth using the SEM images and the Zetasizer measurement in Fig. 1. Mediated by the CES process, the particle size of Cur/MPs with shellac coating gets smaller. The size distribution of the obtained MPs herein would be considered favorable for colitis targeting because of the EPR effect and their ability to resist clearance via diarrhea [35]. More importantly, shellac coating on zein MPs could shield the positive charge of zein, resulting in significant charge reversal from +28.5 to − 25.3 mV. In that manner, the shellac coating procedure is beneficial for avoiding digestion and adherence of zein MPs in the stomach and small intestine. Furthermore, the drug loading and encapsulation efficiencies of Cur loaded in both zein MPs and shellac-coated zein MPs were very high, close to 100%. Therefore, shellac@Cur/MPs exhibited a favorable status for colon-targeting by oral administration, with the suitable particle size and zeta potential.

**Table 1:** Characteristics of Cur loaded MPs (mean ± SD; n = 3)

MPs	Particle size (µm)	Zeta potential (mV)	Specific surface (cm^2^/g)	Drug loading (%)	Encapsulation efficiency (%)
Cur/MPs	4.73 ± 0.18	+ 28.5 ± 0.9	1906.912 ± 3.756	9.98 ± 0.18	98.56 ± 1.23
Shellac@Cur/MPs	2.84 ± 0.15	− 25.3 ± 1.2	2773.356 ± 2.346	8.12 ± 0.19	95.97 ± 3.51

**Fig. 2:**
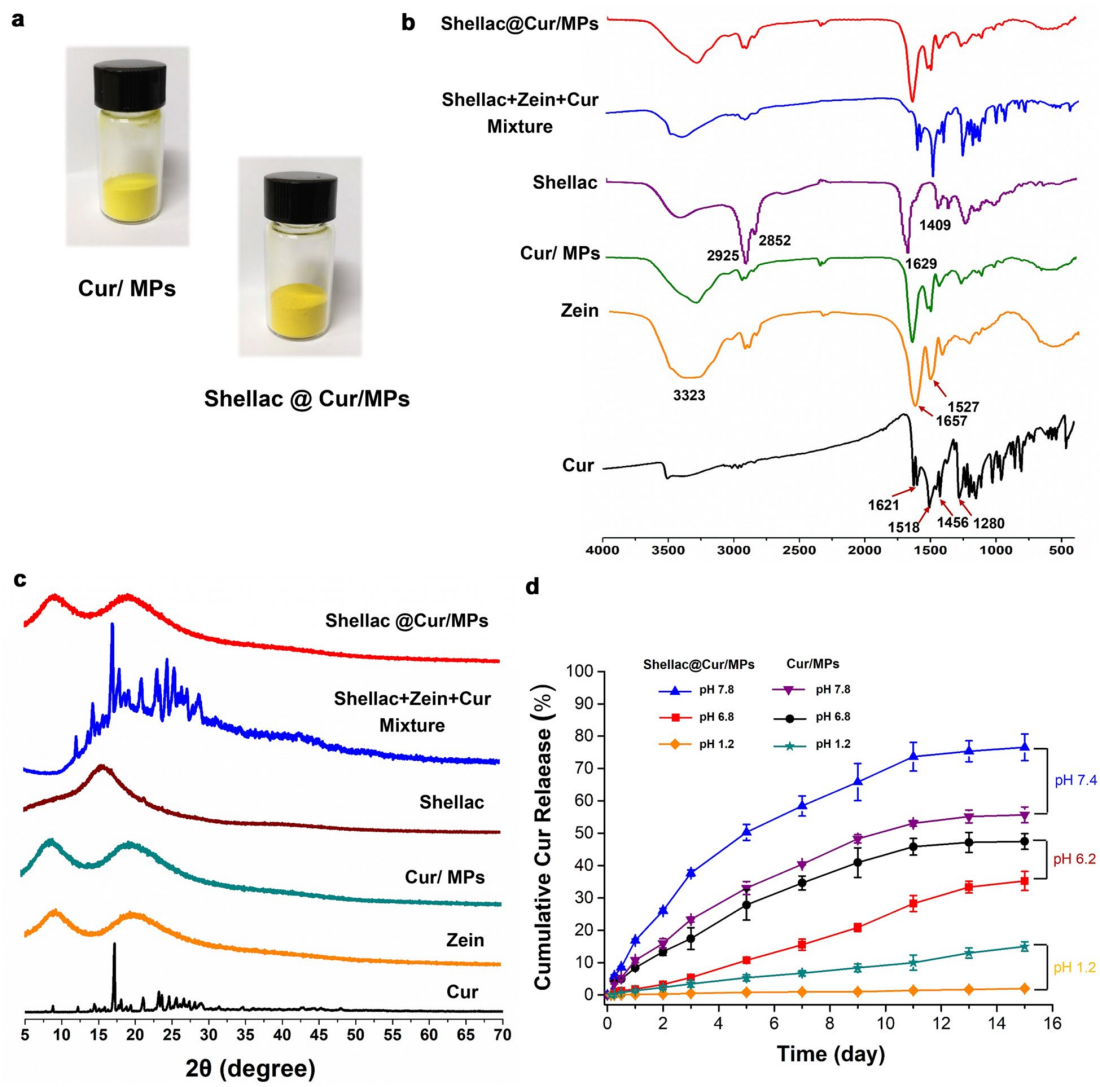
**a** The appearance images of Cur/zein MPs and Shellac coated Cur/zein MPs samples; FT-IR spectrum (**b**) and XRD analysis (**c**) were used to characterize the drug loading in zein MPs and Shellac coated zein MPs. **d** In vitro Cur release profiles of Cur/MPs and Shellac@Cur/MPs in different release medium with pH values to simulate the gastrointestinal tract

Figure 2a shows the appearance of the collected Cur/MPs and shellac@Cur/MPs, exhibiting the yellow powder. Both FTIR and XRD analyses were performed to investigate drug-polymer interactions. In Fig. 2b, some representative absorption bands of free Cur were observed, such as 1621 cm^−1^ (stretching vibrations of benzene ring), 1518 cm^−1^ (C=O and C–C vibrations), 1456 cm^−1^ (olefinic C–H bending vibration), and 1280 cm^−1^ (aromatic C–O stretching vibration) [36]. Meanwhile, there were also some peaks at 880, 852, and 806 cm^−1^ in the fingerprint region [37]. Additionally, Fig. 2b shows the infrared spectrum of zein. Three characteristic bands corresponding to a protein were observed. The peaks at 1657, 1527, and 3323 cm^−1^ corresponded to the vibrational stretch of –C=O, the flexion vibration of the –N–H bond, and the vibration of the –OH and –NH_2_ of zein. All these peaks could be found in the physical mixture of shellac, zein, and Cur, while after loading into zein MPs, these peaks from Cur disappeared. It indicated that the characteristic absorption peaks of Cur related to the aromatic ring and interring chain vibrations were greatly changed after loading into the zein MPs.

X-ray powder diffraction was used to determine the changes in the crystal morphology of Cur in MPs. Fig. 2c shows the sharp crystal diffraction peak of Cur, indicating good crystallinity of the Cur powder. The similar crystal diffraction peaks in the spectrum of physical mixture appeared, demonstrating that the physical mix did not result in the crystal form of Cur. Nevertheless, there were no characteristic diffraction peaks in either Cur/zein MPs or shellac@Cur/MPs, indicating the vastly decreased crystallinity of Cur. This XRD results manifested that the crystallinity of Cur was almost reduced as amorphization in the Cur-loading MPs.

### In vitro* release profiles of Cur from MPs in different mediums*

The controlled drug release profile is an important prerequisite for oral colon-specific preparations for treatment [38]. The *in vitro* release profiles of Cur from MPs in different pH value release mediums were evaluated to the pH-sensitive sustained drug release of shellac@Cur/MPs. As Fig. 2d shows, both Cur/zein MPs and Shellac@Cur/zein MPs exhibited very slow release profiles at pH 1.2, indicating that either zein MPs or shellac-coated zein MPs could prevent drug burst release in the acidic microenvironment of the stomach, owing to the hydrophobicity of zein and shellac materials. The Cur release in Cur/zein MPs and shellac@Cur/zein MPs could be accelerated in simulated intestinal fluid. Particularly, on day 11, the cumulative drug release rate of Cur/zein MPs in pH 6.8 enhanced to about 45%, instead of the 10% release amount of Cur/zein MPs in pH 1.2. Nevertheless, because of the shield of shellac coating, the drug release from shellac@Cur/MPs was more sustained than in the uncoated Cur/MPs. However, Cur loaded in shellac@Cur/MPs got burst release in pH 7.8 medium, indicated the pH sensitivity of shellac material. These results suggested that after loading in shellac-coated zein MPs, Cur exhibited the pH-sensitive sustained drug release.

### Colon distribution and retention

**Fig. 3:**
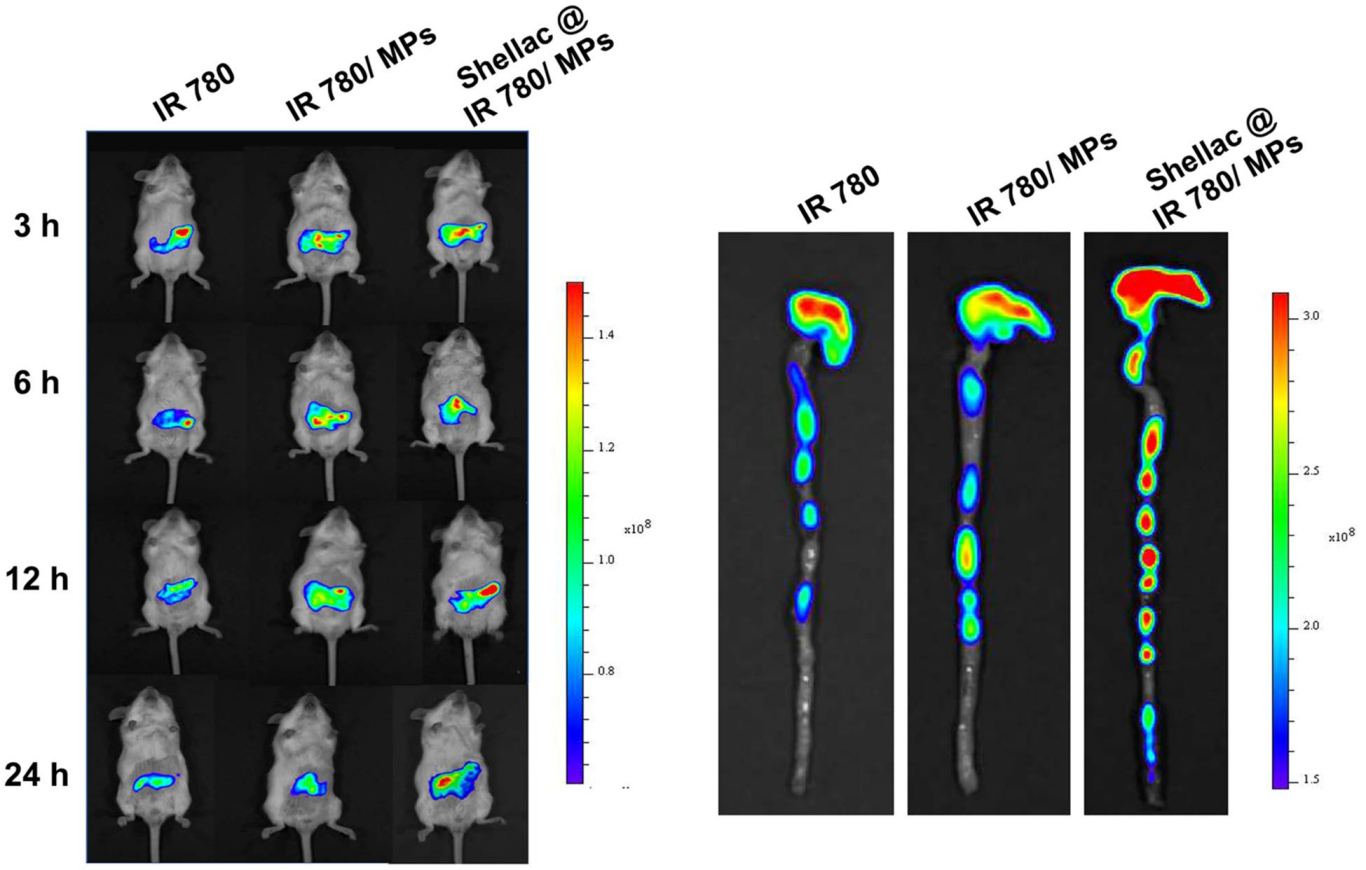
In vivo colon targeting and retention of shellac-coated zein MPs in colitis mice, mediated by the indication of IR780 encapsulation. **a** In vivo fluorescence imaging of the entire colitis mice with oral administration of free IR780, IR780/zein MPs and shellac@IR780/zein MPs at the post-administration 3 h, 6 h, 12 h and 24 h, respectively; **b** At 24 h post-administration, colon tissues in each group were dissected and observed by ex vivo fluorescence imaging

Animal real time living imaging systems can monitor and evaluate the targeting of oral colon-targeting preparations in mice in real time. We encapsulated the near-infrared fluorescent probe IR780 in either zein MPs or shellac@zein MPs to manifest the colon distribution and retention *in vivo*. As shown in Fig. 3a, after oral administration of free IR780 for 24 h, the intestinal fluorescence intensity of mice eliminated rapidly. Nevertheless, the intestinal fluorescence intensity of mice with intragastric administration of shellac@IR780/MPs showed higher residual fluorescence than either IR780/MPs without shellac shield or free IR780, owing to the protection of zein MPs and shellac coating. This result was displayed more distinctly in Fig. 3b by *ex vivo* imaging of the dissected colorectal tissue at post-administration for 24 h. As shown, without the zein MPs loading, most of the fluorescence in the free IR780 group was quenched. Although IR780 loaded in the zein MPs exhibited more residual fluorescence in the colon than free IR780, shellac@IR780/MPs displayed the strongest retention capacity. These results suggested that shellac-coated zein MPs could benefit the colon-targeting for the loaded Cur.

### Efficacy of Cur-MPs in DSS-induced colitis animal model

**Fig. 4:**
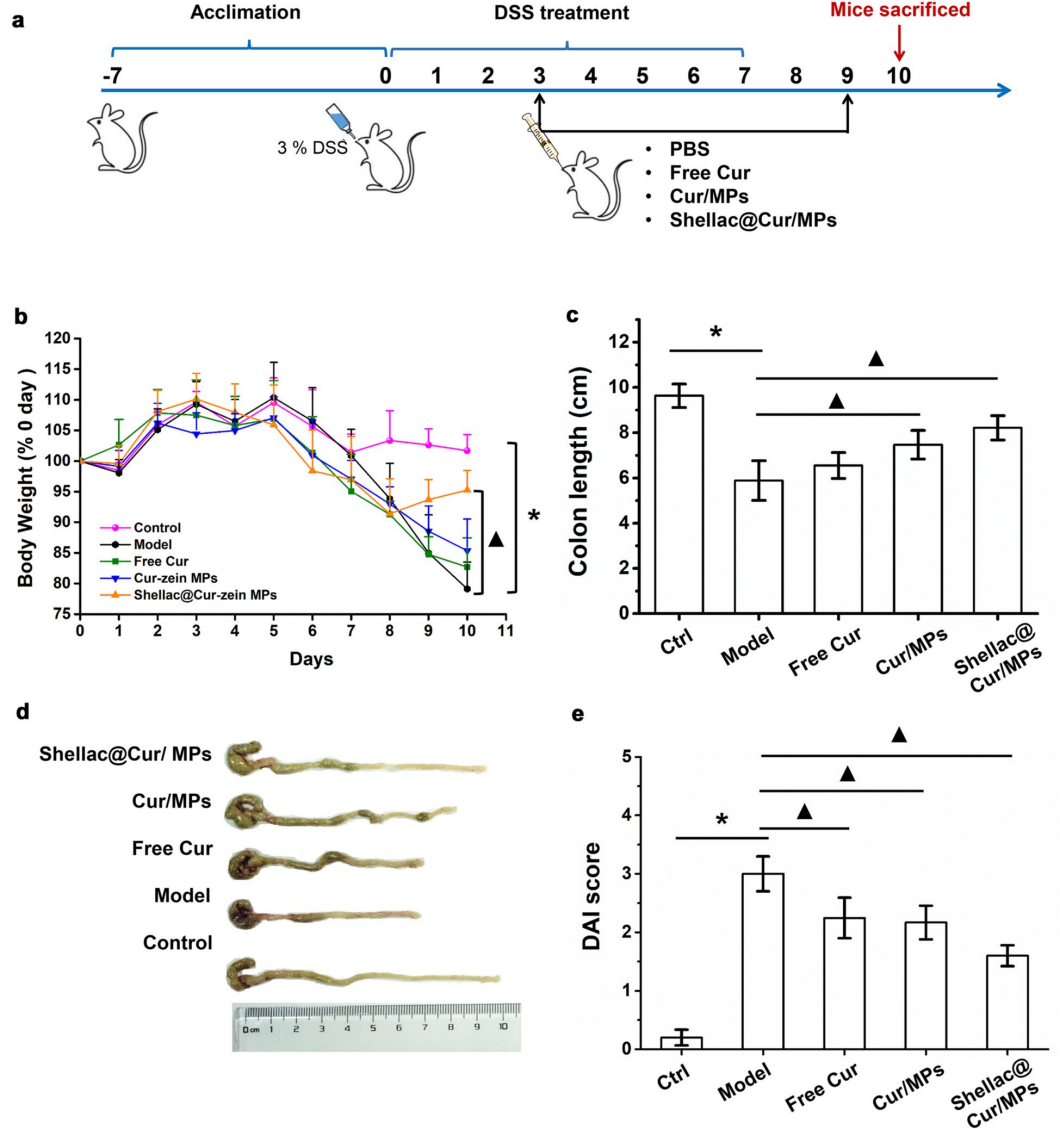
**a** Treatment schedule in mouse bearing DSS-induced colitis. **b** The daily weight change in different group of mice. **c** Colon length of mice in various groups. **d** Representative photographs of mice’s colons in various groups. **e** DAI scores of mice in various groups. *P < 0.05 untreated control group vs. DSS-induced UC model group, ^*▲*^*P* < 0.05 model group vs. treatment groups (n = 6 per group)

The UC mouse model induced by DSS was established to estimate the therapeutic effect of Cur/MPs. We conducted the experiment on the treatment of colitis as per the method of modeling before administration, as shown in Fig. 4a. After the mice were given DSS intervention for 3 days, the mice developed obvious diarrhea and were treated with corresponding Cur related preparations. The body weight changes of mice throughout the experiment clearly reflected their physiological status; this represents one of the main disease features of colitis [5]. As shown in Fig. 4b, the body weight of the mice in the control group fluctuated within a small range and increased steadily. The body weight of mice in the other groups fluctuated greatly under the intervention of DSS, especially the average weight of mice in the model group decreased by 20% on the 10th day compared with the body weight on day 0. After the intervention treatment with corresponding Cur preparation treatment, the weight loss rate of mice did not show any immediate improvement. After discontinuing the free drinking DSS, the body weight of mice in the Shellac@Cur/MPs treatment group showed a recovery increase that normalized compared to the body weight at 0 days, and there was a significant difference compared with that in the model group. However, the body weight of free Cur group and Cur-Zein group mice decreased slightly, but did not increase at an inflection point.

Furthermore, intestinal ulcer, mucosal injury, and bleeding, and inflammation can lead to colorectal shortening. The colonic length of colitis mice is also one of the main evaluation indicators for colitis disease incidence. As shown in Fig. 4c, d, the average colonic length of the controls was 9.8 cm, while that of the model group mice with DSS intervention was averagely shortened to 5.8 cm. Compared to the model group, under the intervention treatment of Cur, the colon shortening of mice in the treatment group was delayed to varying degrees, among which, the treatment effect of Shellac@Cur/MPs group was significantly remarkable, and the colon length of mice tended to normalize; there was a significant difference between the treatment group and the model group.

**Fig. 5:**
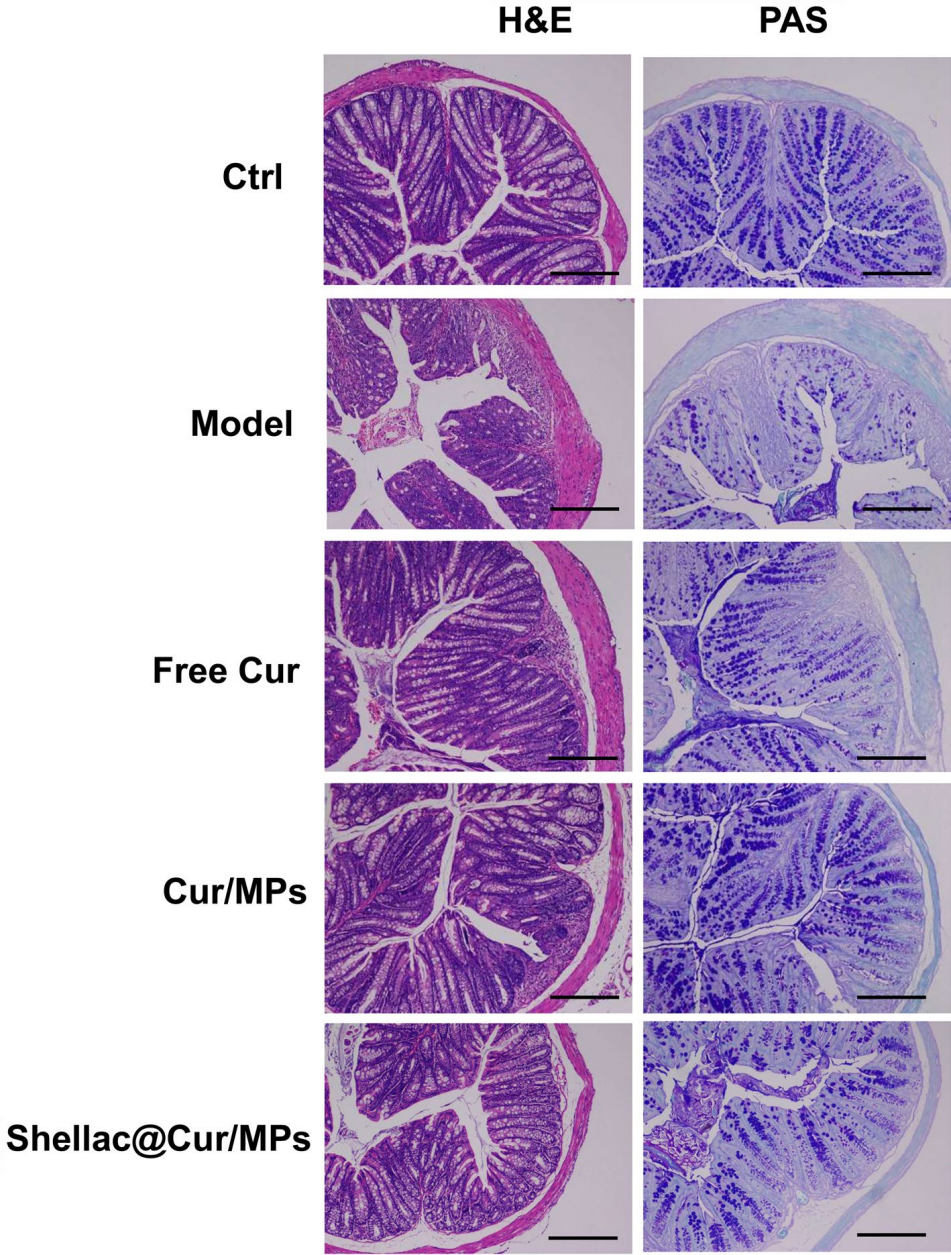
The representative H&E-staining and PAS staining images of colon sections obtained from different groups. The sales at bottom right are 100 μm

In view of the clinical characteristics of these representative, we used the DAI to estimate the therapeutic effectiveness of Cur-MPs. As shown in Fig. 4e, the average DAI score of the model group was significantly higher than that of the control group. However, the DAI scores of Shellac@Cur/MPs dropped remarkably, indicating the improvement effect of Shellac@Cur/MPs on UC-related pathological symptom. Based on the above-mentioned findings, oral DSS can induce severe inflammation of colon tissue. In the DSS-induced model group, the colon tissues showed obvious injuries, including crypt distortion, goblet cell loss, severe epithelial damages, and mucosal inflammatory cell infiltration. However, Shellac@Cur/MPs administration could significantly protect the colon crypt structure and reduce histologic inflammation. Moreover, PAS staining demonstrated that the oral-Shellac@Cur/MPs-treated mice colons possessed a significant proportion of the regenerative colonic crypts with mucus-producing goblet cell morphology (Fig. 5) that was closer to the healthy colon than the colon from mice subjected to colitis.

**Fig. 6:**
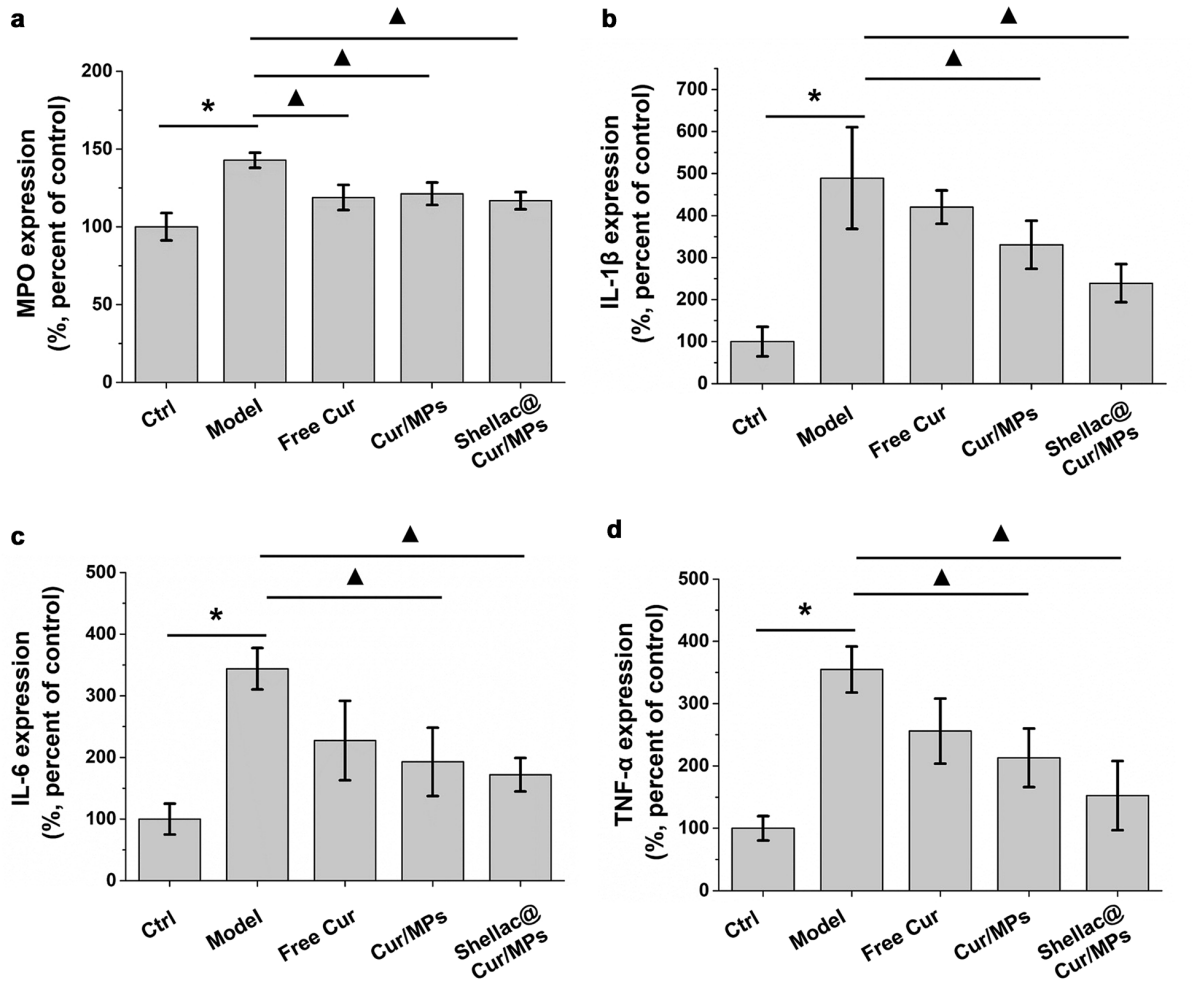
**a** Colonic MPO activity of different groups of mice. **b**, **c** and **d** Relative IL-1 β, IL-6 and TNF-α activity of different groups of mice determined by ELISA. *P < 0.05 untreated control group vs. DSS-induced UC model group, ^▲^*P* < 0.05 model group vs. treatment groups (n = 6 per group)

In intestinal mucosal immune system, macrophages and neutrophils responsible for secretion of inflammatory cytokines, can destroy the integrity of the epithelium, resulting in damage to the colon [39]. MPO is a cellular enzyme released during the migration of multinucleated granulocytes that are mainly produced by macrophages. Its concentration in the colon tissue is an important indicator of colitis severity [39]. As shown in Fig. 6a, the production of MPO was significantly increased in the DSS-induced model group than in the control group. However, with treatment using free Cur and Cur-MPs, the production of MPO in the treatment group showed a downward trend, and there was a significant difference from the model group. As shown in Fig. 6b, c and d, the pro-inflammatory cytokines produced by the DSS-induced model group were significantly higher than those produce by the control group. However, both Cur/MPs and Shellac@Cur/MPs inhibited the abnormally increased amount of pro-inflammatory cytokines. It has been widely confirmed that the rise of MPO, TNF-α, IL-6 and IL-1β is related to the inflammation of mucosa and superficial ulcers in UC mice.

## Discussion

Oral colon-specific drug delivery systems (OCDDS) can deliver drugs directly to the colon and release them, which is a new type of targeted drug delivery system that It is used in the treatment of inflammatory bowel disease, colon cancer, and other diseases. Currently, it has become an important field of targeted drug delivery system research. Although OCDDS can protect the drug from reaching the colon, effective drug release after the drug reaches the colon lesion remains a challenging issue associated with the use of the OCDDS [40]. In this study, Shellac@Cur/MPs with a core–shell structure was prepared using coaxial electrospray. The process is easy to operate, safe, and reliable, and there is no loss of raw materials. The prepared Cur-MPs have high encapsulation efficiency and drug loading, and the use of shellac as a shell material can not only be used as a pH-sensitive material to protect the gastrointestinal delivery of drugs, but also can be used as a sunscreen agent to avoid Cur oxidative decomposition that is beneficial for the formulation preservation. The preparation process provides a reference for the development of MP formulations.

The physical and chemical characterization of the preparation mainly involves particle size measurement, SEM morphology characterization, XRD, and drug release measurement. The test results showed that Shellac-Cur-Zein MPs have a particle size of 2.847 µm, are slightly negatively charged, and have a regular spherical appearance. The XRD test results show that Cur is completely encapsulated in the MPs in an amorphous molecular state. The *in vitro* drug release test shows that Cur can be released stably in the pH 7.4 buffer solution environment and has a certain pH sensitivity.

The efficiency of the colon-targeted delivery affects the efficacy of Cur. In this study, live animal imaging was used to detect the delivery performance of the prepared MPs in the mouse intestine. Shellac coating can effectively protect the formulation from damaging in gastrointestinal tract, so that the immature drug release before it reaches into colon. However, fluorescence imaging reaction drug delivery has certain limitations. Furthermore, we determined the plasma Cur concentrations in UC mice at a seriers of post-administration timepoints (i.e. 0.25, 0.5, 0.75, 1, 1.5, 2, 4, and 8 h) after intragastric administration of various Cur formulations. At 1.5 h post-administration, the plasma Cur concentration in free Cur group reached to the peak value about 0.63 μg/ml. And then, the plasma Cur concentration exhibited to decline rapidly. However, during 8 h post-administration, few Cur could be determined in plasma samples from UC mice administrated with Cur/MPs, and Shellac@Cur/MPs, due to the colon-specific drug delivery. Therefore, although it was difficult to calculate the pharmacokinetic parameters of all these groups, it also corroborated that this system could avoid the pre-mature drug release and absorption in gastrointestinal tract. This result was in keeping with the distribution and retention result mediated by fluorescence dye.

UC is a chronic inflammatory gastrointestinal disease characterized by the destruction of the epithelial barrier and intestinal homeostasis. The latest experimental and clinical data show that pro-inflammatory cytokines such as TNF-α, IL-1β and IL-6 play an important role in the pathogenesis of colitis [41]. In this study, shellac@Cur/MPs exerted anti-inflammatory effects by inhibiting the secretion of pro-inflammatory factors MPO, IL-1β, TN-α and IL-6 in the colon.

In sum, Shellac@Cur/MPs have good biocompatibility, colon localization, pH sensitivity, and can release drugs in specific colon sites and exert therapeutic effects. The preparation can be used for the subsequent development of capsules and tablets, providing a reference for developing Cur preparations.

## Conclusion

The shellac@Cur/MPs were facilely fabricated with the core (zein)-shell (shellac) structure, based on the coaxial electrospray technology, to enhance the oral delivery of Cur and its anti-UC efficacy. The resultant shellac@Cur/MPs had an average hydrodynamic particle size of 2.8 µm, zeta potential of − 25.3 mV, and a high encapsulation efficiency of 95.97%. Mediated by the coaxial electrospray process, Cur was molecularly dispersed within the zein MPs. Based on the protection of shellac-coated zein MPs, Cur could exhibit specifically burst release in artificial colonic fluid and targeted retention in the colon tissue. Particularly, the anti-UC capacity of Cur was significantly enhanced after loading into shellac@zein MPs via inflammation remission. Collectively, it is feasible to prepare core–shell MPs loading Cur with the coaxial electrospray method to improve the therapeutic potency in UC treatment *via* oral administration.

## Data Availability

The data used to support the current study are available from the corresponding author on reasonale request.

## Abbreviations

EPR: Enhanced permeability and rentention effect
PLGA: Poly(lactic-co-glycolic acid)
PBS: Phosphate buffer saline
PAS: Periodic acid-schiff stain
IL-1β: Interleukin-1β
IL-6: Interleukin-6
TNF-α: Tumor Necrosis Factor-α
MPO: Myeloperoxidase
ANOVA: Analysis of Variance
H&E: Hematoxylin and eosin
HPLC: Hight performance liquid chromatography
